# CAR-Toner: an AI-driven approach for CAR tonic signaling prediction and optimization

**DOI:** 10.1038/s41422-024-00936-1

**Published:** 2024-02-14

**Authors:** Shizhen Qiu, Jian Chen, Tao Wu, Li Li, Gang Wang, Haitao Wu, Xianmin Song, Xuesong Liu, Haopeng Wang

**Affiliations:** 1 School of Life Science and Technology, ShanghaiTech University, Shanghai, China; 2 Department of Hematology, Shanghai General Hospital, Shanghai Jiaotong University School of Medicine, Shanghai, China; 3 ENT Institute and Department of Otorhinolaryngology, Eye & ENT Hospital, Fudan University, Shanghai, China; 4 Jiangsu Center for the Collaboration and Innovation of Cancer Biotherapy, Cancer Institute, Xuzhou Medical University, Xuzhou, Jiangsu China; 5 State Key Laboratory of Advanced Medical Materials and Devices, ShanghaiTech University, Shanghai, China; 6 Shanghai Clinical Research and Trial Center, Shanghai, China

**Keywords:** Tumour immunology, Cancer immunotherapy

Dear Editor,

Tonic signaling of chimeric antigen receptors (CARs) plays a pivotal role in governing CAR-T cell fitness: inefficient tonic signaling results in poor CAR-T persistence, while excessive tonic signaling leads to CAR-T exhaustion.^1–3^ Our previous work has elucidated that positively charged patches (PCPs) on the surface of the CAR antigen-binding domain facilitate CAR clustering, thereby triggering CAR tonic signals. To quantify these PCPs, which are indicative of CAR tonic signaling, we previously developed a bioinformatic method to determine the PCP score.^1^ This calculation method starts with constructing three-dimensional (3D) homology models for CAR’s single-chain variable fragments (scFvs) using the SWISS homology modeler. Subsequently, the BindUP web server is used to determine the total count of residues within the top three largest patches containing continuous positively charged residues on the surface of CAR scFv. However, this PCP score calculation method has several limitations: 1. reliance on two external servers; 2. each calculation taking a few days, significantly hindering efficiency; 3. lack of batch calculation capability; 4. no optimization strategies provided for fine-tuning PCP scores. Given these constraints, we aimed to develop an artificial intelligence (AI)-based PCP score calculator and optimizer to overcome these bottlenecks.

**Fig. 1:**
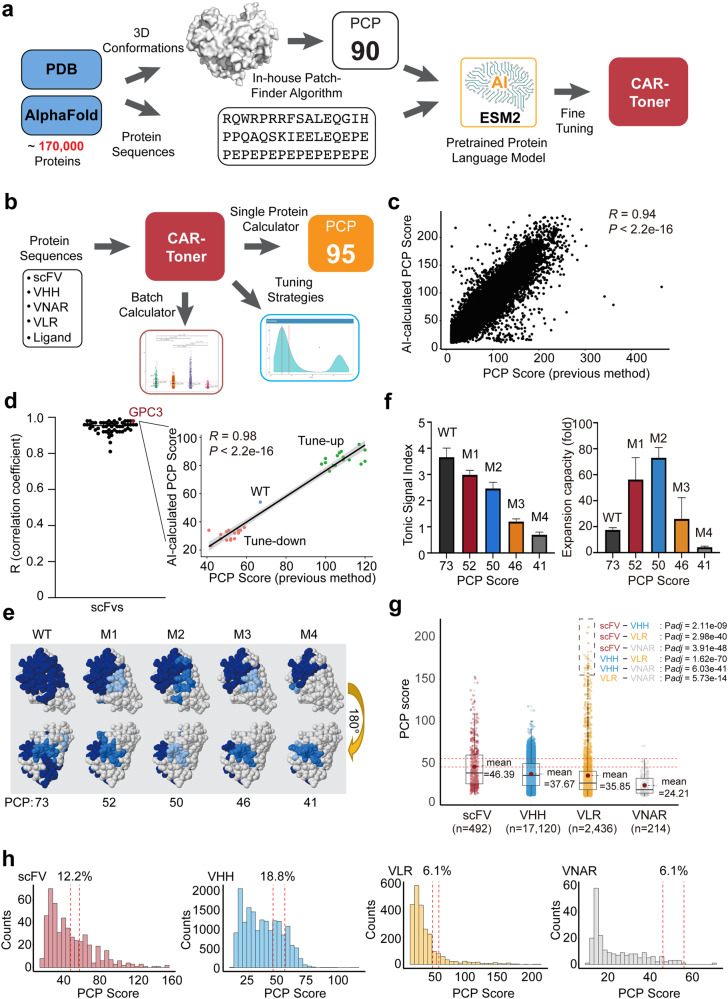
AI-empowered CAR-Toner for predicting and optimizing CAR tonic signaling. **a** Schematic diagram illustrating the development process of CAR-Toner. **b** Three major functions of the CAR-Toner: after inputting the sequences of the CAR antigen-binding domain, CAR-Toner can calculate the PCP of single or batch-inputted sequences and offer solutions for PCP optimization for the protein provided. **c** Correlation between the AI-predicted PCP scores of protein sequences from the testing database using the CAR-Toner and their PCP scores calculated by the previous approach assessed by the Pearson method.^1^ **d** Correlation coefficient of 54 scFvs tested using CAR-Toner. **e** Display of PCPs on CLL1 variant antibody surface displayed by the BindUP web server tool. Dark blue, first-largest PCP; medium blue, second-largest PCP; light blue, third-largest PCP. **f** Tonic signaling indexes and the expansion capacities of CAR-T cells incorporating WT and mutated CLL1 antibodies with various PCP scores. **g** Batch calculations of PCP scores for scFv, VHH, VNAR, and VLR datasets using the CAR-Toner. **h** Proportion of antibody or antibody alternative sequences with PCP scores between 46 and 56 in the dataset of scFv, VHH, VNAR, and VLR, respectively. All comparisons were determined using Student’s *t*-tests.

Protein databases, structural biology, and advanced deep learning models are all integrated into our AI-based PCP score calculator (Fig. 1a). A comprehensive protein structure database consisting of over 170,000 entries was established by extracting 3D structural information from the Protein Data Bank (PDB) and AlphaFold predictions, followed by stringent quality control procedures. We further developed an in-house algorithm tailored for calculating PCP scores based on the obtained 3D structure information (Supplementary Information), subsequently generating a dataset comprising approximately 170,000 protein sequences along with their associated PCP scores. For model training and evaluation, 70% of the data are allocated as the training dataset, while the remaining 30% serve as the test dataset. The ESM2 model, developed by the FAIR (Meta Fundamental AI Research Protein Team), is utilized for fine-tuning tasks related to PCP prediction.^4,5^ ESM2 is a transformer-based language model using an attention mechanism to learn interaction patterns between pairs of amino acids in the input sequence. Pre-trained on over 60 million protein sequences from the UniProt Reference Clusters (UniRef) database, ESM2 demonstrates strong adaptability to downstream protein structure-related tasks.^5^ The ESM2-8M model was used to fine-tune the training dataset. Following updating parameters, the ESM2 model was transformed into the PCP-AI prediction model, referred to as CAR-Tonic Signal Tuner (abbreviated as CAR-Toner; http://cart-fitness.slst.shanghaitech.edu.cn/CAR-fitness/). This model encompasses three key functionalities: proficient PCP calculation for individual proteins, streamlined batch processing, and an integrated optimization strategy for refining PCP scores (Fig. 1b).

CAR-Toner demonstrated accurate performance on the testing dataset (*R* = 0.94, *P* < 2.2e^*−*16^, Fig. 1c). To further assess the proficiency of its PCP calculations, we collected 54 CAR scFv sequences from previous studies (Supplementary information, Table S1) and compared AI-calculated PCP scores with PCP scores calculated using the previous method. A significantly linear correlation was observed (*R* = 0.85, *P* < 2.2e^*−*16^, Supplementary information, Fig. S1). CAR-Toner can also robustly tune up or down the PCP scores of these scFvs by inducing point mutations (Supplementary information, Fig. S2). Among CAR variants of each scFv generated by the CAR-Toner, we observed a strong linear correlation coefficient (Fig. 1d), further demonstrating the proficiency of the CAR-Toner.

To investigate the potential of CAR-Toner in optimizing CAR design, we selected a camelid single-domain nanobody (VHH) against CLL1, a tumor-associated antigen of acute myeloid leukemia (AML), as an example. CAR-Toner analysis revealed a high PCP score of 73 for this CLL1 antibody, correlating with strong tonic signaling, high exhaustion level and low expansion capacity (Fig. 1e, f; Supplementary information, Fig. S3a). We systematically tuned down its PCP score, generating a series of CLL1 mutants with gradually decreasing PCP scores (Fig. 1e). As expected, we observed that reduced PCP scores were associated with decreased CAR tonic signaling and ameliorated CAR-T exhaustion (Supplementary information, Fig. S3a). Intriguingly, in an ex-vivo proliferation assay, a progressive decline in PCP scores initially enhanced CAR-T cell expansion, peaking around PCP scores of 50–52, and then reducing sharply around PCP score of 40 (Fig. 1f). This aligns well with the PEAK theory we proposed previously, suggesting that an intermediate tonic signaling strength optimally benefits CAR-T cell function.^1,2^ Of note, the M2 mutant, with its finely-tuned tonic signaling, exhibited improved cytotoxicity compared to the wild-type (WT) CAR (Supplementary information, Fig. S3b). Collectively, our results suggest that CAR-T cells with optimized tonic signaling through our AI-based tool exhibit improved functional performance and that PCP influences the tonic signaling of nanobody-based CAR-T cells, similar to its impact on scFv-based CAR-T cells.

There are various types of antibodies and antibody alternatives commonly utilized as the antigen-binding domains of CARs, including scFv, VHH, shark single-domain nanobody (VNAR) and variable lymphocyte receptors derived from jawless vertebrates (VLR).^6–8^ We aimed to compare the PCP features of these four types of CAR antigen binding domains, taking advantage of CAR-Toner’s batch calculation capacity. We assembled a large collection of these protein sequences (scFv, *n* = 492; VHH, *n* = 17,120; VNAR, *n* = 2436; VLR, *n* = 214) for CAR-Toner analysis (Fig. 1g). Compared to other antigen-binding domains, we found that VNAR has an exceptionally low PCP score (mean value = 24.21), indicating that VNAR-based CAR-T cells likely lack persistence. Notably, a unique proportion (~1%) of VLRs have extremely high PCP scores (> 155), suggesting that these VLR-based CAR-Ts are likely to become exhausted. Our previous study has shown that the optimal PCP score for generating CAR signaling to support superior T cell fitness is estimated to be around 46–56.^1^ Our results indicate that, among the four types of antigen-binding domains, VHH-based CARs have the highest proportion within this optimal range (Fig. 1h). Overall, these data suggest that VHH nanobodies, with their intrinsic PCP feature aligning CAR-T fitness, might be a high-quality antigen binder for CAR-T development.

In summary, we developed CAR-Toner, an AI-based tool for PCP calculation. It also provides optimization recommendations for PCP scores; yet, the precise impact of these optimizations on CAR antigen-binding specificity and affinity necessitates further validation. Nonetheless, this innovative tool is poised to drive forward the CAR-T design field towards the next generation, paving the way for AI-powered advancements in CAR-T design.

### Supplementary information

Supplemental material of CAR-Toner: An AI-Driven Approach for CAR Tonic Signaling Prediction and Optimization
