# Supplementary material for: CAR-Toner: an AI-driven approach for CAR tonic signaling prediction and optimization
Source: Cell Res. 2024 Feb 14;34(5):386–8. doi: 10.1038/s41422-024-00936-1 (PMC11061301; doi:10.1038/s41422-024-00936-1)
Supplement: Supplementary file 1 — Supplemental material of CAR-Toner: An AI-Driven Approach for CAR Tonic Signaling Prediction and Optimization [file 41422_2024_936_MOESM1_ESM.pdf]

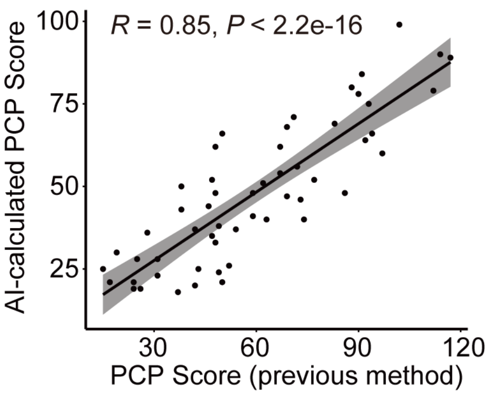

Fig. S1 Compare the PCP Scores calculated by CAR-Toner and our previous method.

Correlation between the PCP score calculated by AI and the previous method.

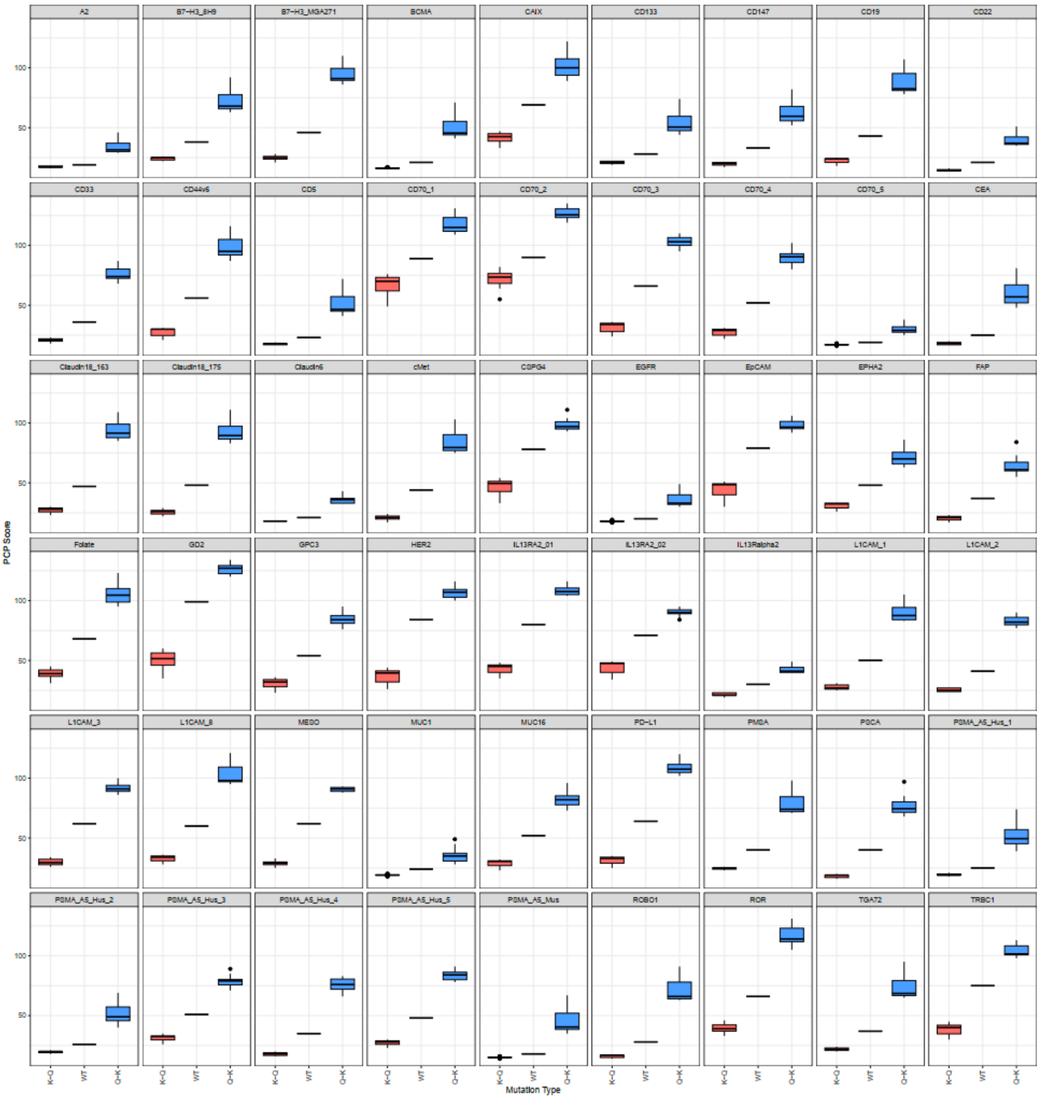

Fig. S2 Modulation of PCP Scores across 54 CAR Sequences using AI-powered CAR-Toner.

To increase PCP scores, the neutral amino acid (Q) was mutated to the positively charged lysine (K), and the reverse for decreasing scores. For each CAR sequence, up to five amino acid residues were targeted, constructing variants with combinations of 3, 4, or 5 mutations per sequence. For instance, mutating 3, 4, or 5 out of five selected residues resulted in 10, 5, and 1 mutated CAR variants, respectively. The distribution of PCP values of CAR mutants for these 54 CAR sequences was illustrated. Red: decreased PCPs; Black: WT-CAR PCP; Blue: increased PCPs.

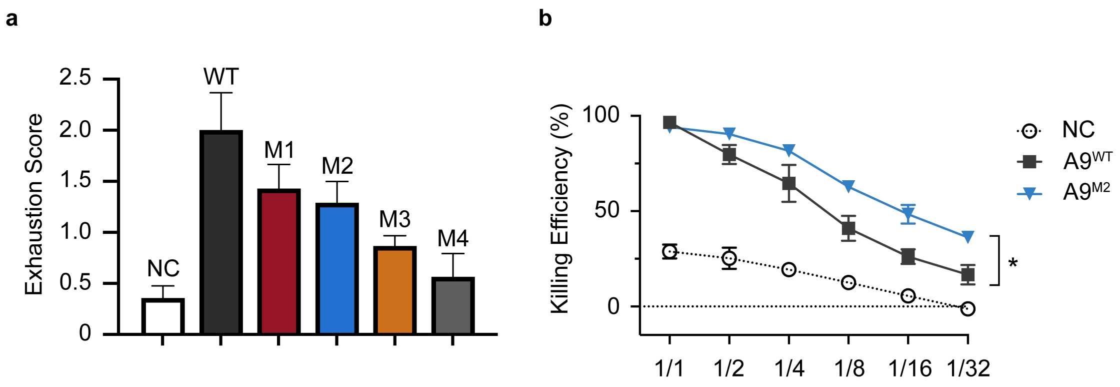

Fig. S3 Exhaustion levels and cytotoxic capabilities of CLL-1 CAR-T variants.

a Primary T cells were transduced with different CAR variants. The exhaustion score was determined as described previously. In brief, the exhaustion score was calculated by taking the average expressions of three typical exhaustion markers: PD-1, Tim-3, and Lag-3.

b The indicated CAR-T cells were co-incubated with their target tumor cells at the indicated E:T ratio overnight. The killing efficiency was analyzed using a luciferase cytotoxicity assay.

Number	name	cor	Number	name	cor	Number	name	cor
1	A2	0.95	19	CSPG4	0.96	37	L1CAM_8	0.91
2	B7-H3_8H9	0.96	20	Claudin18_163	0.98	38	MESO	0.91
3	B7-H3_MGA271	0.96	21	Claudin18_175	0.96	39	MUC1	0.9
4	BCMA	0.92	22	Claudin6	0.93	40	MUC16	0.98
5	CAIX	0.94	23	EGFR	0.94	41	PD-L1	0.97
6	CD133	0.95	24	EPHA2	0.97	42	PMSA	0.97
7	CD147	0.93	25	EpCAM	0.95	43	PSCA	0.97
8	CD19	0.96	26	FAP	0.95	44	PSMA_A5_Hus_1	0.92
9	CD22	0.98	27	Folate	0.94	45	PSMA_A5_Hus_2	0.81
10	CD33	0.92	28	GD2	0.97	46	PSMA_A5_Hus_3	0.97
11	CD44v6	0.98	29	GPC3	0.98	47	PSMA_A5_Hus_4	0.96
12	CD5	0.89	30	HER2	0.97	48	PSMA_A5_Hus_5	0.96
13	CD70_1	0.86	31	IL13RA2_01	0.98	49	PSMA_A5_Mus	0.92
14	CD70_2	0.92	32	IL13RA2_02	0.96	50	ROBO1	0.95
15	CD70_3	0.95	33	IL13Ralpha2	0.92	51	ROR	0.93
16	CD70_4	0.97	34	L1CAM_1	0.93	52	TGA72	0.96
17	CD70_5	0.95	35	L1CAM_2	0.98	53	TRBC1	0.96
18	CEA	0.93	36	L1CAM_3	0.99	54	cMet	0.98

Table. S1 54 scFv used to compare the PCP score calculated by AI and the previous method. The table shown the 54 scFvs uesd to test the correlation coefficient using CAR-Toner or our previous method .

Methods

Reagents and antibodies

Cell culture medium and the supplements, including FBS, were purchased from Thermo Scientific. DAPI (D9542) and sodium chloride (S5886) were from Sigma. Human T-Activator CD3/CD28 Dynabeads (11132D) were from Life Technologies. Recombinant human IL-2 (GMP-CD66) was from Novoprotein. The following antibodies were used for fluorescence-activated cell sorting: anti-CD69-PE (1:800, Biolegend, 310906), anti-CD25-PerCP-Cy5.5 (1:800, Biolegend, 302625), anti-ICOS-PE-CY7 (1:800, Biolegend, 313520), anti-Myc-Alexa Fluor 647 (1:1000, Cell Signaling Technology, 2233S).

Cell lines

Lenti-X 293T was purchased from TaKaRa. Jurkat T cell line and CLL1+ THP-1 Acute Myelocytic Leukemia cell line were kindly gifted from A.Weiss at UCSF and Xianmin Song at Shanghai General Hospital of ShanghaiJiaotong University School of Medicine, respectively. The adherent cells were cultured in DMEM medium while suspension cells were maintained in RPMI-1640 medium, both of which were supplemented with 10%FBS, 100 U/mL penicillin and 100 mg/mL streptomycin.

CAR construction and retrovirus production

The generations of CAR plasmids and lentivirus were conducted as reported in our previous work. In short, each CAR comprised of a specific VHH, a CD28 costimulatory domain, a CD3ζ signaling domain, and an IRES-EGFP cassette was cloned into a modified PHR vector with the hEF1α promoter. Point mutations for CARs were performed via PCR and Gibson assembly cloning. Lentivirus supernatants were generated in Lenti-X 293T cells with the CAR plasmid and viral packaging plasmids.

Transduction and expansion of human T cells

Human peripheral blood mononuclear cells (PBMCs) from healthy donors were cultured in x-vivo medium(Lonza, 04-418Q) supplemented with 2% FBS, 100 U/mL penicillin, 100 mg/mL streptomycin，0.292mg/mL glutamine, and 200 U/mL recombinant hIL-2. Before transduction, T cells were stimulated with Human T-Activator CD3/CD28 Dynabeads for 24 h first. On the next day, T cells were incubated with the virus for 18 hours before medium replacement. Medium containing hIL-2 was refreshed every 2-3 days. Dynabeads were removed on day 4. In certain cases, sodium chloride was added to the medium after transduction. Cells used for experiments were harvested about 2 weeks after transduction.

Flow cytometry

For cell-surface staining, cells were incubated with antibodies at 4 °C for 30 min in the dark. In most cases, the Zombie Violet Fixable viability Kit was used to exclude dead cells. Samples were acquired on LSRFortessa and analyzed with FlowJo v.10 software.

In vitro proliferation assay

The proliferative ability of CAR-T cells was assessed as in our previous work. Briefly, 1×10^6 CAR-T cells were stimulated by irradiated tumor cells (E:T = 1:1) for 3 days after a full rest without hIL-2. Cell number was counted every other day by Trypan Blue dye exclusion, and the cell culture density was kept at 1×106 T cells per ml with fresh medium meanwhile.

In vitro cytotoxicity assay

Targeted tumor cells were engineered to stably express the firefly luciferase firstly. The luciferase-expressing tumor cells were seeded at 5 × 10^4 cells/well in 96-well plates and incubated with various numbers of CAR-T cells at multiple E:T ratios for 24 h at 37 °C. Subsequently, the co-culture system of each well was transferred to a specific luminometer plate, and 0.15 mg D-luciferin was added afterward. PerkinElmer Enspire was used to observe the luminescence intensity of each well immediately. The killing efficacy was calculated by normalizing the luminescence intensity of coculture wells to that of tumor cell monoculture.

Calculation of tonic signaling index and exhaustion score

To measure the tonic signaling index, primary T cells were transduced by CARexpressing virus. After GFPpositive cells were gated, the mean fluorescence intensity (MFI) of CD69-PE and MFI of GFP in these cells were measured. CAR tonic signaling index was calculated using the following formula:

CAR tonic signaling index = MFI^{CD69}/MFI^{GFP}

To calculate the exhaustion score, the surface expression of PD-1, Lag-3 and Tim-3 in these GFP-positive cells was determined by FACS. CAR exhaustion score was assessed using the following formula:

CAR exhaustion score = $(MFI^{PD\text{-}1}/MFI^{GFP} + MFI^{Lag\text{-}3}/MFI^{GFP} + MFI^{Tim\text{-}3}/MFI^{GFP}) / 3$

Data sources

For the experimentally obtained protein structure, protein sequence FASTA files and protein structure PDB files were downloaded from the RCSB PDB database[1] . As we focus on predicting PCP score of antigen-binding domains of CARs, we screened for proteins with single-chain structures and sequence lengths between 100 and 400. Duplicated sequences were further removed, then PCP values were calculated for the remaining protein structures. Protein sequences with PCP greater than 10 were filtered and finally 13,256 sequence-PCP matching data were obtained. For the calculated protein structure, protein sequence and 3D structure files were downloaded from Uniprot[2] and Alphafold[3] databases, respectively. The Alphafold was used for predicting the structure of a single protein chain with a naturally occurring sequence. Thus, we only need to select proteins with a length between 100 ~ 400. After removing duplicated sequences, calculating PCP scores, and filtering PCP scores, more than 10,156,339 sequence-PCP matching data were generated. The training and testing data of the model is obtained by combining the above two parts of data. Commonly used CAR scFv sequences were obtained from the FPO website (https://www.freepatentsonline.com).

In-house PCP calculation algorithm

Similar to the PatchFinder algorithm[1] , Pdb-tools[4] was used to remove all HETATM records in the PDB file, which record nonstandard residues. Hydrogen atoms are added prior to the calculations using pdb2pqr30[5]. Then, the electrostatic potential is calculated using the APBS[5] software with a grid spacing of 1Å. The threshold of 2 kT/e was applied to filter out positive electrostatic potential grid points. We further define the grid points that fall on the protein surface, using the DMS open source to calculate the surface accessibility, based on the Lee and Richards algorithm[6] , ignoring all non-surface points. We then extract continuous patches by selecting all 3D patches of adjacent grid points by cut-off 1Å. The PCP value is obtained by summing the number of residues in the first three largest patches. These procedures are implemented in the Python programming language.

Model Fine Tuning

168917 sequences were randomly divided into training sets and test sets in a ratio of 7:3. The training set was used to train the model and update parameters, while the test set was used to verify the performance of the model. The ESM2 (https://github.com/facebookresearch/esm) pre-trained model was chosen as the base model. ESM2 contains a series of pre-trained models with different parameter sizes (from 8M to 15B). For ease of training and deployment, we selected the smaller ESM2-8M model. ESM2-8M has 6 Transformer layers, 8 million parameters, and the amino acid embedding vector length is 320 dimensions. In order to adapt to the PCP prediction task, we added a fully connected layer at the end of the pre-trained model so that the output dimension is 1 to fine-tune the regression task. The model hyper-parameters are: learning rate: 0.0002, training epoches: 15, weight decay: 0.001, optimizer: adamw. After fine-tuning was completed, the model's weights were saved for subsequent predictions. Fine-tuning was implemented by Transformers library of huggingface.

Web interface

For the convenience of users, we built a web interface to calculate PCP and provide an optimization strategy of antigen-binding domains of CARs based on PCP score. The web interface of CAR-Toner consists of two parts: single-mode and batch-mode. In single mode, the user can input one sequence and calculate its PCP score. Then, the system shows the number of mutated sequences and the position of tuning mutations (k-q for tuning down, q-k for tuning up). Once the user clicks the “tuning” button, the PCP prediction of mutated sequences is running. After completion, the tuning results are shown as a table and the distribution of PCP scores are also drawn. Users can select a specific range of PCP scores in the density plot and corresponding results are listed in the table below. In batch mode, the user can input multiple sequences in the text box or in the FASTA file, and the predicted results are shown as a table.

Code available

All code for data processing, model training and construction of shiny-app are available at https://github.com/wt12318/PCP-AIOptimizer.

Reference

1. Paz, I., et al., *BindUP: a web server for non-homology-based prediction of DNA and RNA binding proteins.* Nucleic Acids Res, 2016. **44**(W1): p. W568-74.
2. UniProt, C., *UniProt: the Universal Protein Knowledgebase in 2023.* Nucleic Acids Res, 2023. **51**(D1): p. D523-D531.
3. Jumper, J., et al., *Highly accurate protein structure prediction with AlphaFold.* Nature, 2021. **596**(7873): p. 583-589.
4. Rodrigues, J., et al., *pdb-tools: a swiss army knife for molecular structures.* F1000Res, 2018. **7**: p. 1961.
5. Jurrus, E., et al., *Improvements to the APBS biomolecular solvation software suite.* Protein Sci, 2018. **27**(1): p. 112-128.

6. Lee, B. and F.M. Richards, *The interpretation of protein structures: estimation of static accessibility.* J Mol Biol, 1971. **55**(3): p. 379-400.
